# Supplementary figures and images for: CD40L protects against mouse hepatitis virus-induced neuroinflammatory demyelination
Source: PLoS Pathog. 2021 Dec 13;17(12):e1010059. doi: 10.1371/journal.ppat.1010059 (PMC8699621; doi:10.1371/journal.ppat.1010059)

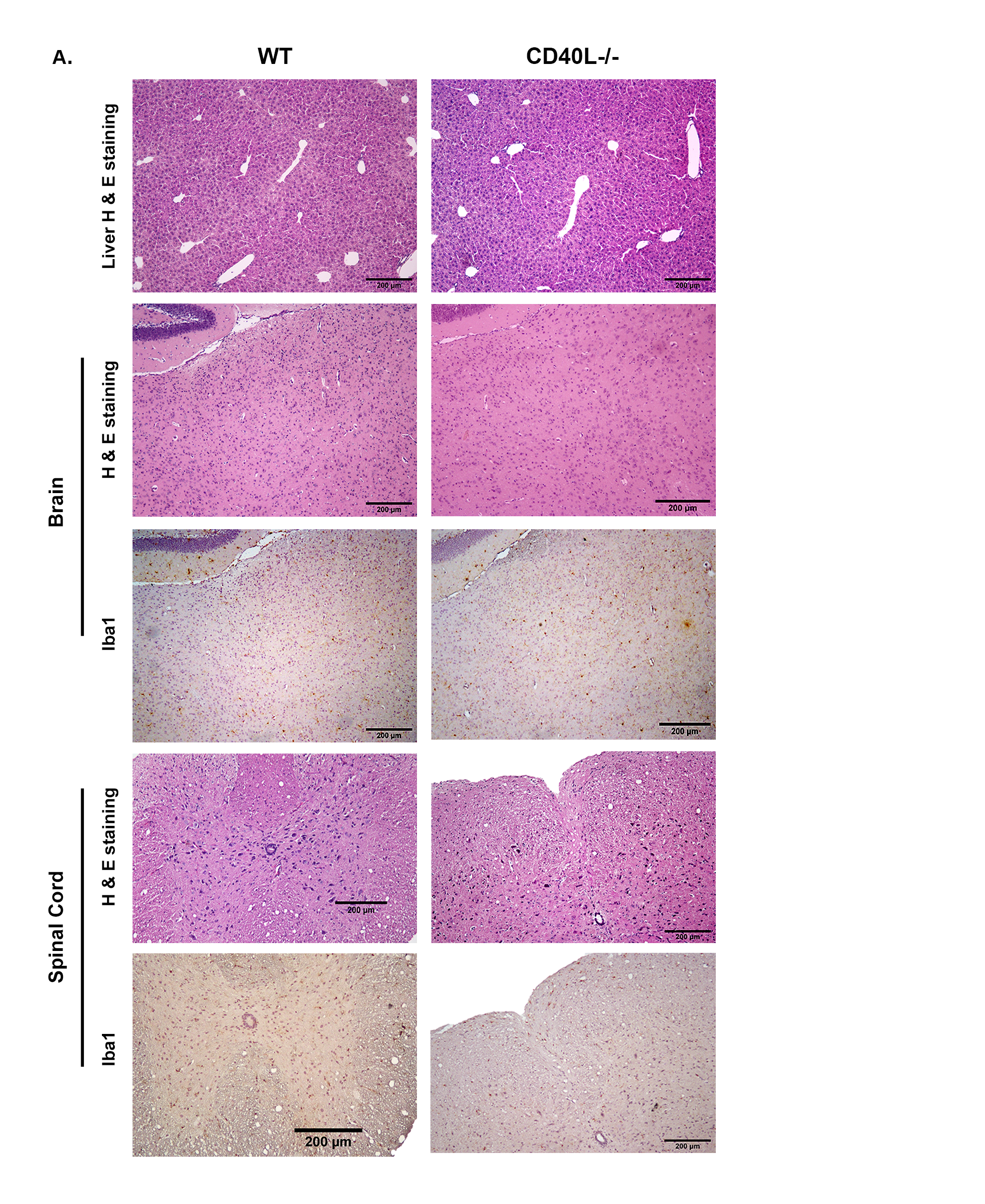

Supplement: S1 Fig — WT and CD40L-/- mice were infected with an uninfected cell lysate (PBS + 0.75% BSA). A. Five-micrometer-thick tissue sections were stained with H&E (liver, brain, and spinal cord) and anti-Iba1(brain and spinal cord) for routine histopathological studies. No significant inflammation was observed in WT and CD40L-/- mouse tissues. The data represents results from 3 independent biological replicates. Scale bars, 200μm. (TIF) [file ppat.1010059.s001.tif]

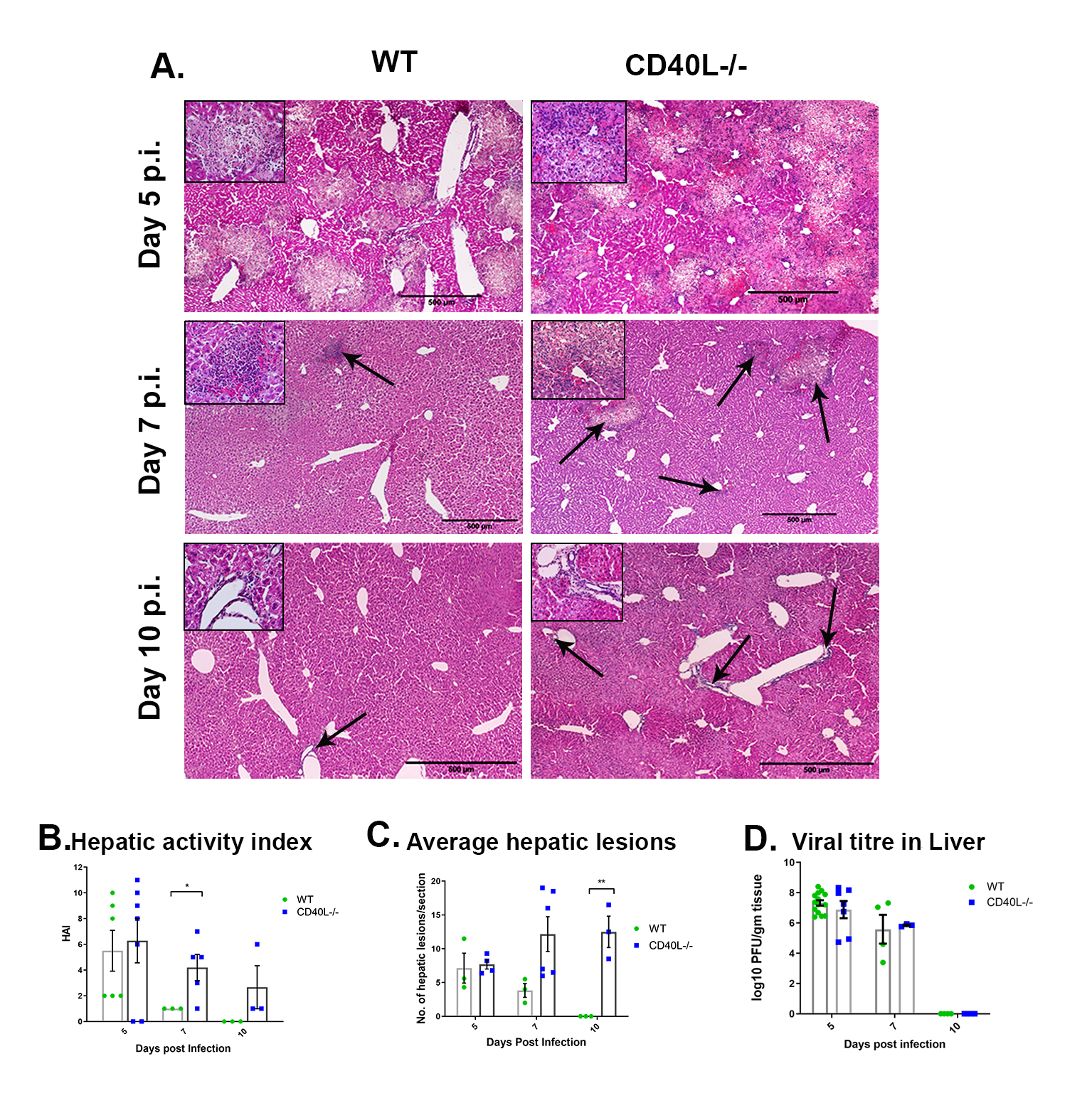

Supplement: S2 Fig — RSA59 infection is known to cause acute mild-moderate hepatitis. High mortality in CD40L-/- mice at a clinical score of less than 3.5 (moribund) and significantly high weight loss, thus suggested an investigation of systemic inflammation in the liver. (A) WT and CD40L-/- mice (N = 4–5 per time point) were subjected to histopathological analyses of liver tissues by H & E staining. Arrows show hepatic lesions, manually enlarged lesions are depicted in insets. (B) The hepatic activity index was calculated according to Ishak’s score, as described in Materials and Methods, and plotted. (C) The average numbers of hepatic lesions per section from each mouse were determined, and the combined results were plotted. (D) Viral titer was determined in the liver homogenates on day 5, 7 and 10 p.i. and plotted. Results were expressed as mean ± SEM. *Asterisk represents statistical significance calculated using unpaired Student’s t-test, p<0.05 was considered as significant. *p<0.05, **p<0.01. Scale bar 500 μm. (TIF) [file ppat.1010059.s002.tif]

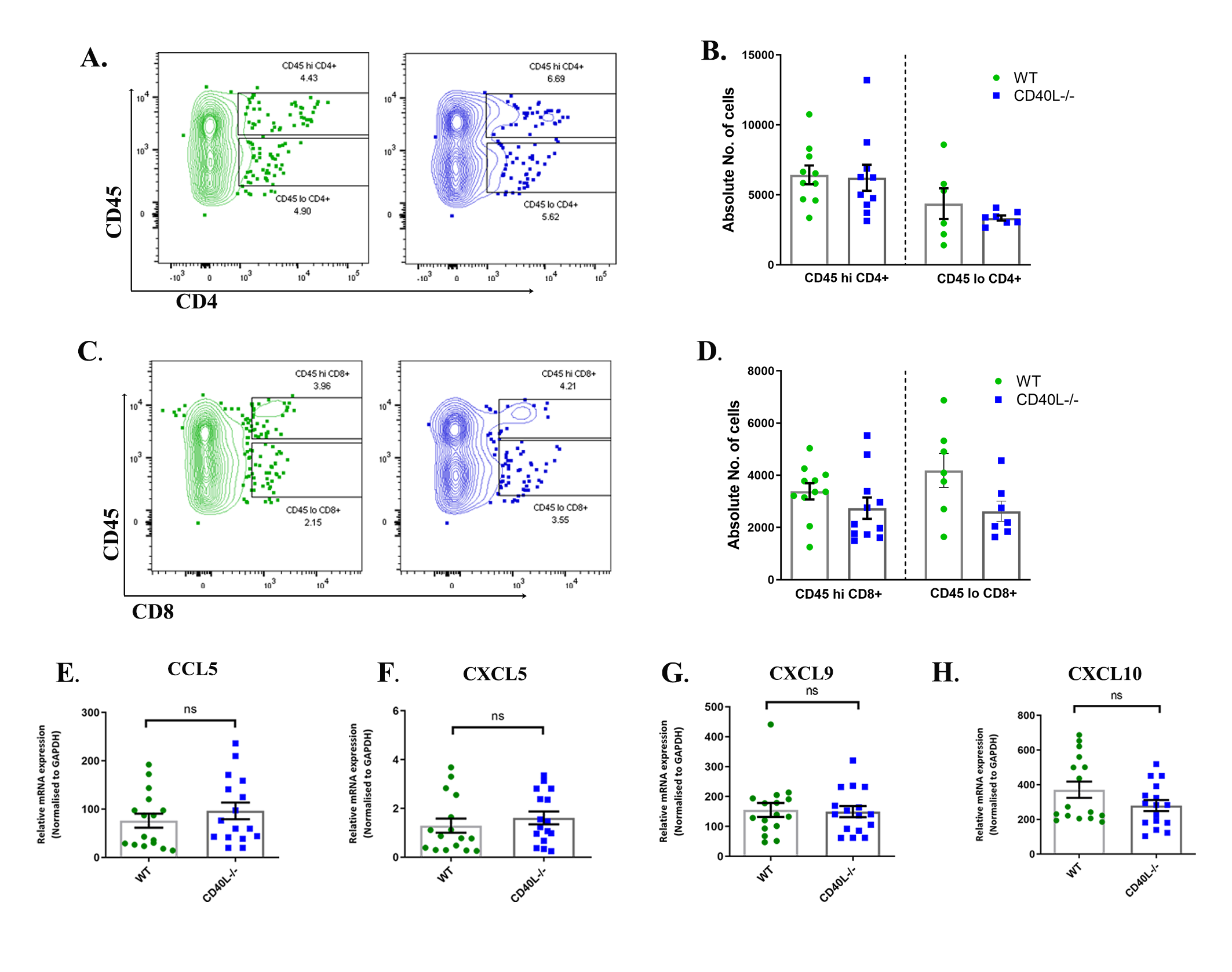

Supplement: S3 Fig — On day 5 p.i. brains from WT and CD40L-/- mice infected with RSA59 were harvested for flow cytometry analysis and stained for CD45, CD4, and CD8. The green color denotes WT, and blue indicates CD40L-/- mice. Primary gating was performed on singlets followed by live cells and CD45. A, B. Representative flow cytometry plots indicating percentages and graphical representation of absolute numbers of CD45hiCD4+ and CD45loCD4+ comparing WT and CD40L-/- groups. C, D. Representative flow cytometry plots showing the percentages and graphical representation of absolute numbers of CD45hiCD8+ and CD45loCD8+ comparing WT and CD40L-/- groups. RSA59 infected brains of WT and CD40L-/- mice were subjected to qRT-PCR analyses of chemokines, E. CCL5, F. CXCL5, G. CXCL9, and H. CXCL10. Results were normalized to GAPDH, compared with mock-infected control, and expressed as mean ± SEM from 3 independent biological experiments (N = 3–5). Statistical significance was calculated using unpaired Student’s t-test and Welch correction, p<0.05 was considered significant. (TIF) [file ppat.1010059.s003.tif]

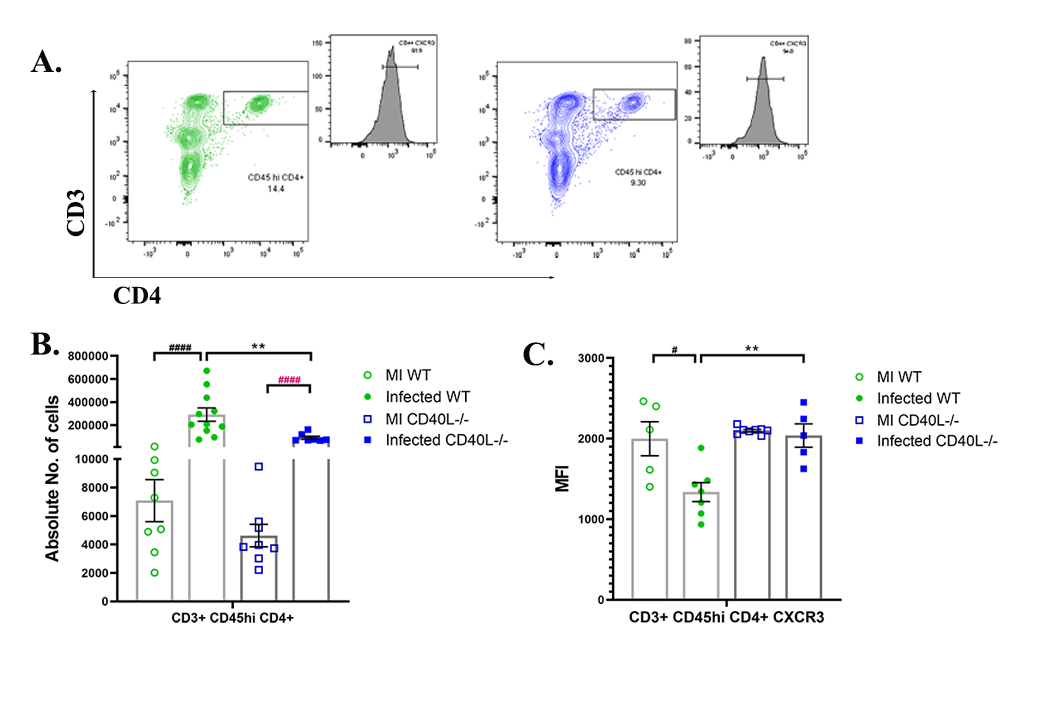

Supplement: S4 Fig — On day 10 p.i., brain-derived cells from MI and RSA59 infected (10000 PFUs) WT and CD40L-/- mice were harvested for flow cytometry analysis and stained for CD3, CD45, CD4, and CXCR3. The green color denotes WT, and blue indicates CD40L-/- mice. Primary gating was performed on singlets, followed by live cells and CD3 and CD45. (A) Representative flow cytometry contour plots indicating percentages of CD45hiCD3+CD4+ and histograms showing CD3+CD45hiCD4+CXCR3+ cells from infected sets. (B) Graphical representation of absolute numbers of CD3+CD45hi cells expressing CD4 and (C) Median Fluorescence Intensity of CXCR3 expression on CD3+CD45hiCD4+ cells comparing MI and infected WT and CD40L-/- groups. Results were expressed as mean ± SEM from 3 independent biological experiments (N = 4). *Asterisk (MI WT v/s infected WT) and #hash (black-infected WT v/s infected CD40L-/-, pink- MI CD40L-/- v/s infected CD40L-/-) represents statistical significance calculated using unpaired Student’s t-test and Welch correction, p<0.05 was considered significant. **p<0.01, **p<0.01, ****pp<0.0001. (TIF) [file ppat.1010059.s004.tif]

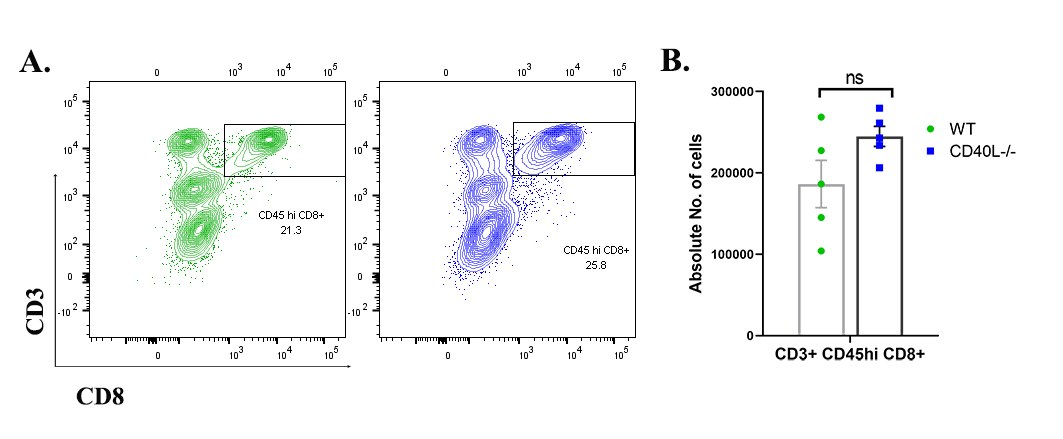

Supplement: S5 Fig — On day 10 p.i. brains from WT and CD40L-/- mice infected with RSA59 were harvested for flow cytometry analysis and stained for CD45, CD3 and CD8. The green color denotes WT, and blue indicates CD40L-/- mice. Primary gating was performed on singlets followed by live cells, CD45 and CD3. A, B. Representative Flow cytometry plots showing the percentages and graphs showing the absolute numbers of CD45hiCD3+CD8+ comparing WT and CD40L-/- mice groups. Results were expressed as mean ± SEM from 3 independent biological experiments (N = 3). Statistical significance calculated using unpaired Student’s t-test and Welch correction, p<0.05 was considered significant. (TIF) [file ppat.1010059.s005.tif]

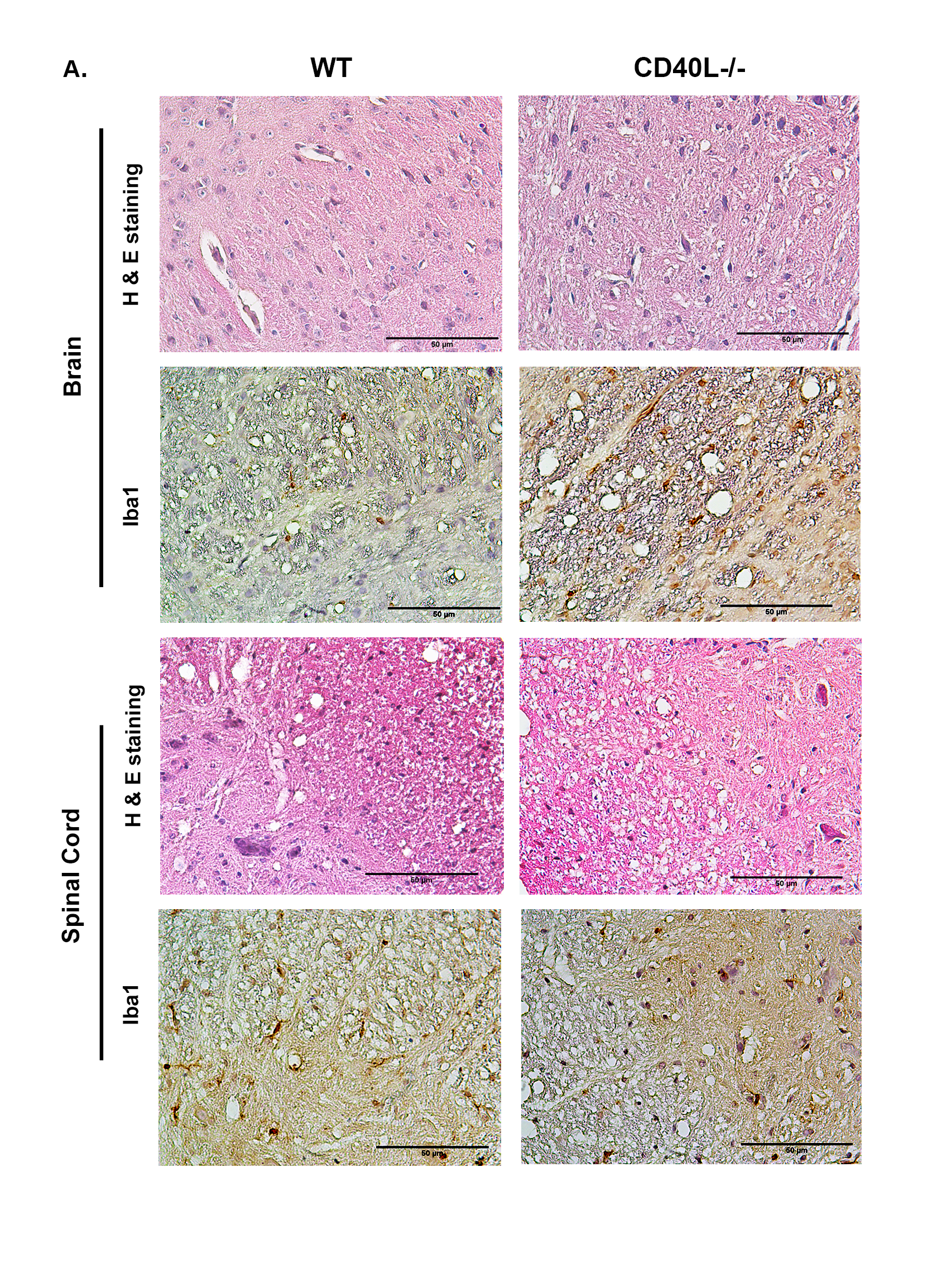

Supplement: S6 Fig — WT and CD40L-/- mice were infected with an uninfected cell lysate (PBS + 0.75% BSA). A. Five-micrometer-thick tissue sections were stained with H&E (brain, and spinal cord) and anti-Iba1(brain and spinal cord) for routine histopathological studies. No significant inflammation was observed in WT and CD40L-/- mouse tissues. The data represents results from 3 independent biological replicates. Scale bars, 50μm. (TIF) [file ppat.1010059.s006.tif]

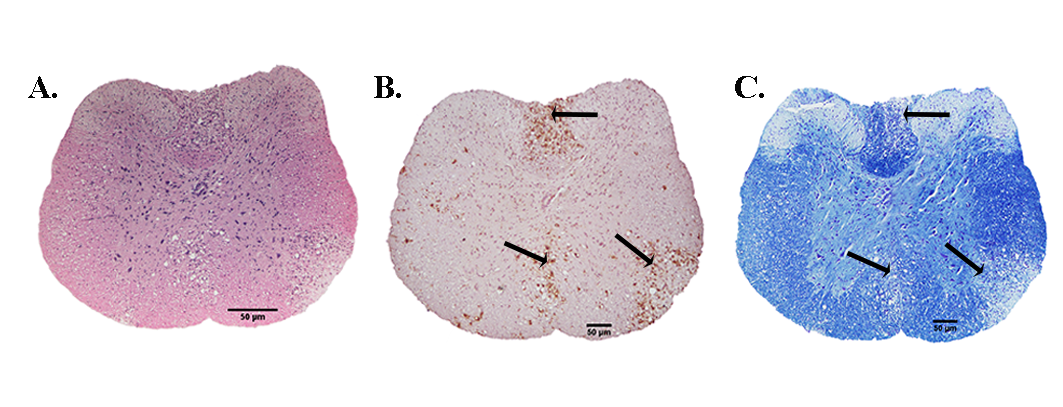

Supplement: S7 Fig — 5μm thick serial cross sections from RSA59 infected WT mice spinal cords at day 30 p.i. were analyzed for A. presence of inflammatory lesions by H&E, B. inflammatory cells by anti-Iba1 (microglia/macrophage) by immunohistochemistry, and C. demyelination by LFB. Black arrows show Iba1+ microglia/macrophages in B and corresponding demyelinating plaques in C. The data represents results from 2 independent experiments (N = 3). Scale bars, 50μm. (TIF) [file ppat.1010059.s007.tif]
