# Supplementary material for: CD40L protects against mouse hepatitis virus-induced neuroinflammatory demyelination
Source: PLoS Pathog. 2021 Dec 13;17(12):e1010059. doi: 10.1371/journal.ppat.1010059 (PMC8699621; doi:10.1371/journal.ppat.1010059)
Supplement: Attachment — Submitted filename: Response to Reviewers_PPATHOGENS-D-21-00896 _09022021.doc [file ppat.1010059.s008.doc]

Dear Dr. Thiel,

We would like to thank you for your kind consideration and allowing us to resubmit the manuscript. We would also like to thank you for sending us the editorial and reviewers’ comments.

We appreciate the Reviewers for their thoughtful and critical comments on our manuscript and we are grateful for your consideration to revising our work for PLOS pathogens. As per the editorial comments and the reviewers’ suggestions we have incorporated the changes in the revised manuscript. In the following sections we have provided point-by-point responses to the issues raised by the reviewers to the best extent possible by doing additional experiments as well as the reanalysis of the data as suggested. All the changes have been highlighted in the track change mode in the revised manuscript. The increase in the N numbers have been specified in the figure legends in the manuscript to the best extent possible, as suggested by the reviewers.

As per the reviewers’ suggestions, we have performed additional experiments to study clinical severity, mortality and weight loss in mice, we have performed viral titers from the liver tissues on days 5, 7, and 10 p.i., as this virus is voraciously known to infect the liver in the periphery, along with the liver histopathology from the same infected mice (that was previously added in the supporting file), but now has been added in the main manuscript. Also, as suggested by the reviewers, additional flowcytometry experiments were performed with mock infected controls both from WT and CD40L-/- mice at days 5, and 10 p.i. and were compared among themselves as well as with the infected mice from both groups. The infected mice group data were also added with the existing data to increase the number of mice infected per group per experiment. In addition, mock-infected controls were also added with the histopathology data for day 30 p.i. in the main manuscript. But unfortunately, due to the lack of CD4 Knockout mice transportation, co-adoptive transfer of genetically marked and CFSE labeled WT and KO CD4 T cells and comparing their responses and migration in WT and CD40L -/- mice could not be performed and are beyond the scope of this manuscript at present.

In order to address the reviewers’ suggested experiments and comments, **the total number of figures in the revised manuscript equals 13 and 3 in the supporting information.** If publishing criteria requires us to reduce the numbers, we can move a few figures to the supporting information.

**Response to Reviewer's Questions**

**Part I – Summary:**

**Reviewer #1:**

In this study, Fareeha Saadi et al. show that mice deficient in CD40L are more susceptible than WT to neuroinflammatory demyelination induced by intracranial infection with mouse hepatitis virus. This is shown at 5, 10 and 30 days post infection after measuring a variety of parameters including virus loads, histopathology innate and adaptive cell infiltration in the brain, etc.

We thank the reviewer for critically reviewing our manuscript and the enthusiasm they showed in our manuscript. Their concerns have been addressed pointwise in the following sections.

**Figure 2A requires more extended time of observation and statistical validation for the claim of difference in mortality.**

We agree with the reviewer that adding the experimental data from different time post infection beyond day 10 will enhance the claim of difference in mortality. But considering the human endpoint (as per the animal ethics) observed in CD40L-/- mice at 25,000 PFUs of RSA59 on day 10 p.i. with severe clinical disease corresponding to both hind limb paralysis and impaired food intake, we could not take the experiments further. But we have conducted few more experiments (WT: N=4, CD40L-/-: N=6) and added the data along with statistical testing. The results were statistically significant by Log-rank (Mantel-Cox) test. (Figure 3 in the revised manuscript)

For further experiments on day 10 and day 30 p.i. were performed at a viral dose of 10,000 PFUs and all mice survived at this dose.

**Most figures lack MI control (3, 4, 5, 6, 7, 8, 9, 10) and this is particularly important in Figure 7.**

The MI control of Figure 3 was already shown in Figure S1 of the old manuscript. But now we have added this figure in the main manuscript as suggested by the reviewer. (Figure 2 in the revised manuscript)

The MI control results have been added in the graphs of Figures 4, 5, 6 and 7 in the revised manuscript Figures 6, 7, 8 and 9.

The MI controls corresponding to Figures 8 and 9 have also been added in the revised main manuscript (Figure 10) as suggested by the reviewer.

**In many micrographs, a greater magnification would help to see the differences.**

We agree with the reviewers, that the high magnification images will help to illustrate our findings better. So, we have added the high magnification images (40X) in Figure 5 of the revised manuscript, below our original 10X images. Though we have added the high magnification images, we have kept the original 10X images, as we aim to represent the differences in the overall presence of Iba1+ microglia/macrophage in the brain. Additionally, we also wanted to highlight the differences in the characteristics of encephalitis that is perivascular cuffing and microglial nodule formation which are both scarce and less in CD40L-/- mice. These overall observations are well represented at 10X (Figure 5).

As suggested by the reviewers, we have also added high magnification (40X) images for Figure 8 and Figure 9, i.e., Figure 11 and 12 in the revised manuscript.

Figure 11 shows the chronic phase inflammation in the brain. As per reviewers’ suggestions, we have added magnified images for the Iba1 staining, highlighting the morphology of microglia/macrophages as well as for the PLP staining highlighting the loss of PLP and axonal blebbing in the lateral white matter tracts in the brain stem regions.

Figure 12 shows the chronic phase inflammation and demyelination in the spinal cords. Higher magnification image corresponding to Iba1 staining has been added.

Some conclusions cannot be made. For example, CD4 T cell priming. More importantly, the data does not support that the increased disease in the C40L-/- mice is due to CD40L deficiency in CD4 T cells. This would require:

**1) Co-adaptive transfer experiment of CFSE (or similar stain) labeled WT and CD40L deficient CD4 T cells.**

**2) Reconstitute CD4 deficient mice with CD40L deficient or WT CD4 T cells.**

We thank the reviewer for their thoughtful comments. It will be very interesting to see whether CD40L is expressed in the subset of lymphoid or myeloid peripheral immune cells other than CD4+ T cells. In parallel, abundant previous literature reports in different systems show that besides CD4+ T cells, CD40L expression is observed in platelets which are not known to interact with the CD40 expressing macrophages (including tissue-specific variants such as brain microglial cells or liver Kupffer cells), dendritic cells and B cells. Therefore, it could be postulated that the effect of CD40L-deficiency is contributed by the CD4+ T cells. This can also be supported by our data showing the significant downregulation of CD40L expression in CD4-/- mice compared to WT upon RSA59 infection (Figure 1E). Also, in this manuscript our major focus is to understand the communication between CD4+ T cells and microglia/macrophages, therefore the suggested experiments are beyond the scope of this manuscript at present. Furthermore, we agree that tracking of CD4+ T cells’ differential trafficking to the site of infection in WT and CD40L-/- mice will be interesting and will provide insightful data, which may further strengthen our findings. However, given the corona pandemic and the new normal, procuring reagents and CD4 knockout mice in a timely manner is not possible, which makes the adoptive transfer experiments beyond the scope of this manuscript.

But we sincerely thank the reviewers for their insightful comments, and we are planning to incorporate these experiments in our future studies.

**Reviewer #2:**

This is a very nicely done study by Saadi et al. examining the role of CD40L in the neurological damage following mouse hepatitis virus (RSA59) infection. The authors show that deficiency in CD40L results in increased neurological disease, which is associated with more severe pathology in early and late stages of disease as well as a decrease in CD4 T cells, microglia cells as well as monocyte/macrophages/neutrophil population (which was not sorted out). Long term damage was associated with prolonged virus in the brain. As the pathogenesis of virus-induced demyelination and damage to the CNS is still a strong area of interest and the authors study the interaction between the innate and adaptive immune response, this paper is of substantial interest to readers of PLOS Pathogens.

We thank the Reviewer for appreciating the design of the work and identifying the study of substantial interest to readers of PLOS Pathogens. Their kind support and thoughtful suggestions will improve our manuscript.

Concerns have been addressed point-wise in the major and minor issues sections.

**Reviewer #3:**

The Saadi et al. study describes the protective role of CD40L expressing CD4+T cells in reducing Mouse Hepatitis Virus-induced neuroinflammatory demyelination. The paper is well written, and the experiments were well-planned. The authors performed a time course experiment assessing the brain and the cervical lymph node at different stages of MHV infection in wild-type and CD40L-/- mice, focusing on examining the molecular interaction between CD4+ T cells and microglia/macrophages. The lack of CD40L leads to increase in demyelination of axons, highlighting the protective role of this interaction. This result contrasts with the well-documented pathogenic role of the CD40-CD40L in an autoimmune disease model, thereby signifying this study.

We thank the Reviewer for appreciating the planning and design of experiments and identifying the study as a significant contribution in the understanding of inflammation and immunomodulation in a virus induced model of Multiple sclerosis.

Concerns have been addressed point-wise in the minor issues sections.

**Part II – Major Issues: Key Experiments Required for Acceptance:**

**Reviewer #1:**

This paper is lacking in mechanism. It would be much improved with:

1) Co-adoptive transfer of genetically marked and CFSE labeled WT and KO CD4 T cells and comparing their responses and migration in WT and CD40L -/- mice.
2) Show that the disease is recapitulated in CD4 deficient mice reconstituted with CD40L-/- mice while they are protected when given WT CD4 T cells. In addition, the authors should see that all figures have MI control mice.

We strongly agree with the reviewer that this co-adoptive transfer will be a very good experiment but is beyond the scope of the manuscript, as explained in detail in the summary section.

As per the reviewer’s suggestion, and mentioned previously, all the MI controls have been added in the main revised manuscript.

**Reviewer #2:**

1) One of the main questions of concern is what happens to the microglia population in the CNS of CD40L-/- mice. The number of resident brain microglia should not be changed dramatically unless the cells are undergoing apoptosis or are actively replicating. Do the numbers of microglia in WT mice increase compared to mock-infected animals? Alternatively, are the microglia in CD40L-/- mice dying, this could be determined by staining tissue sections for Iba1 and active Caspase 3.

**Response 1:** We thank the reviewer for highlighting this important and critical question. We have now included mock infected controls (both WT and CD40L-/-) throughout our experiments. Since, mock infected controls of WT and CD40L-/- mice did not show any significant differences in microglia/macrophage populations in histopathology previously, we did not show the flowcytometry data for mock infected. However, now in our revised main manuscript, we have included the mock infected controls for flow cytometry experiments in all the figures (Figures 6, 7, 8 and 9 in the revised manuscript) in the main manuscript, as per reviewers’ suggestions. Our re-analyzed and new data showed that indeed, microglia numbers (CD45lo CD11b+) increase upon infection in WT mice compared to mock infection including their MHCII, CX3CR1 and CD40 expression. However, interestingly in CD40L-/- mice, infection does not induce any significant increase in the number of CD45lo CD11b+ CNS resident microglia. Additionally, no significant infiltration of CD45hi CD11b+ Ly6G- monocyte/macrophages was observed in CD40L-/- mice upon infection which was significantly more in WT mice (also shown previously).

Due to the area constraint in the figure, we have added the mock data in the graph, but dot plots have been shown only for the infected sets. If reviewers suggest, we can add the mock dot plots in the supplementary figures.

This context has been added in the results as well as the discussion section (Page 6 Line 208-218, Page 7 Line 239-245, Page 11 Line 397-402)

2) The authors do not mention peripheral virus loads in the WT versus CD40L mice. It would be expected that they would be comparable based on the comparable loads in the brain at 5 and 7 dpi, but this should be addressed.

**Response 2.** We have performed viral titers for liver tissues on day 5, 7 and 10 p.i. and included the results in Figure 4 D in the revised manuscript. Results showed no significant difference on day 5 and day 7 p.i. and viral titers were below the detection limit on day 10 p.i. in WT mice (as expected) and viral titers were also below the detection limit in CD40L-/- mice. (Page 6 Line 186-189). This figure was previously included in supporting information as Figure S2 and is now moved to the main manuscript.

3) The experiment in Fig. 2 ends right when mice are developing clinical disease (day 10). The authors need to provide an explanation on why that time frame was not extended further. Was it due to the high level of weight loss in the CD40L-/- mice? Were any of the mice taken out past day 10?

**Response 3:** The rationale for not extending the studies beyond day 10 p.i. has been explained above. But again, to reiterate, considering the human endpoint (as per the animal ethics) observed in CD40L-/- mice at 25,000 PFUs of RSA59 on day 10 p.i. with severe clinical disease corresponding to both hind limb paralysis, impaired food intake, and significant weight loss by 30%, we could not take the experiments further. But as per another reviewer’s suggestions, we have conducted few more experiments (WT: N=4, CD40L-/-: N=6) and added the data along with statistical testing. The results were statistically significant by Log-rank (Mantel-Cox) test. (Figure 2, Page 5 Line 171-185)

For all further experiments on day 10 and day 30 p.i. were performed at a viral dose of 10,000 PFUs and all mice survived at this dose.

**Reviewer #3:**

None

**Part III – Minor Issues: Editorial and Data Presentation Modifications**
Please use this section for editorial suggestions as well as relatively minor modifications of existing data that would enhance clarity.

**Reviewer #1:**

1. Line 46. Please define better the model. Introduce the virus, then explain the route of administration.

**Response 1**: In the revised manuscript, suggested changes have been addressed. (Page 2 Line 48-61)

“A neurotropic of Mouse Hepatitis Virus, MHV-A59) or an isogenic recombinant strain RSA59 infection-induced neuroinflammatory demyelination in an experimental model that typically initiates with the activation of CNS-resident neuroglial cells[1]. A schematic representation (Figure 1 A) depicts the pathogenesis of intracranial RSA59 infection of 4 week old C57BL/6 mice along with the conventional kinetics of viral RNA, infectious virus particles, and the course of RSA59 induced acute neuroinflammation and chronic progressive demyelination. Days 1-7 p.i. are characterized by an onset of neuroinflammation predominated by innate-immune responses, followed by a switch to protective adaptive immunity where acute inflammation resolves, viral titer goes below the detection limit (by routine plaque assay), and viral RNA persists at very low abundance (days 7-15 p.i.). The gradual increase in myelin loss with or without axonal loss starts as early as day 7 p.i., as observed, and reaches its peak at chronic phase of neuroinflammation i.e. day 30 p.i. (Figure 1 A)”

2. Lines 51-55. This is unclear. The model is about chronic infection but then talks about protective immunity. Please explain the model well and possible outcomes.

**Response 2:** In the revised manuscript, suggested changes have been made. (Line)

MHV-A59/RSA59 infection is used as an experimental model to understand direct neural cell dystrophy mediated acute neuroinflammation which is responsible for bringing the peripheral immune cells for viral clearance and ameliorating the pro-inflammatory condition by promoting an anti-inflammatory milieu. But, as this virus upon intracranial inoculation follows axonal route, reach to the spinal cord and release at the nerve end to infect oligodendrocytes at the acute phase, and activate white matter microglia/macrophages in stripping myelin from the damage axonal, thus culminating in myelin loss at the chronic phase.

Thus, to clarify this model, as mentioned in the previous comment (Page 3 Line 77-81)

3. Line 110. Indicate route and pfu.

**Response 3:** In the revised manuscript, suggested changes have been made. (Page 4 Line 134-136)

“Age-matched 5 weeks old C57BL/6 wild-type (WT) and CD4-/- and CD40L-/- male mice in the C57BL/6 background were inoculated intracranially with isogenic enhanced green fluorescent protein (EGFP)-expressing RSA59 at 25000 PFUs for studies on day 5 p.i. and 10000 PFUs for days 7,10 and 30 p.i.”

4. Figure 1A. Please describe in the introduction.

**Response 4:** Suggested changes have been made and this section is included in the introduction to describe the model which also answers the concerns raised in point 1. (Page 2 Line 48-61)

5. Line 132. From the data, it cannot be concluded that CD4 T cells are the primary population expressing CD40L. CD4 T cells could be involved in the recruitment of CD40L expressing cells.

**Response 5:** We agree with the reviewers that CD4 T cells may not be the primary population secreting CD40L. In Figure 1E, we have shown that the CD40L expression is significantly downregulated in CD4-/- mice compared to WT. Considering the reviewers’ suggestion we have rephrased our results as, *Thus the results implicate that CD4+ T cells significantly express CD40L in the brain upon RSA59 infection* (Page 5 Line 154-158)*.*

6. Lane 145. Death of CD40L-/- mice does not seem to be less than 60%. Please indicate if there are significant differences or not in survival. Ideally, mice should be observed for a period of time, until death stabilizes.

**Response 6:** Upon performing additional experiments, we have found the significant difference in mortality between WT and CD40L-/- mice, based on Mantel-Cox test, as mentioned above. The rationale for not extending the studies beyond day 10 p.i. due to the human end point, has been explained above. (Figure 2, Page 5 Line 171-185)

7. Lane 171 should say Figure 3B.

**Response 7:** We thank the reviewer for pointing this out. We have changed it to Figure 3B. (Figure 5B in the revised manuscript, Page 6 Line 202)

8. Line 177. Indicate references demonstrating that CD45 intensity distinguishes cell origin.

**Response 8:** We thank the reviewer for suggesting. We have added the reference. (ref 41)

**Reviewer #2:**

1. As the dose of inoculating virus changes between some experiments, it should be included in the figure legend.

**Response 1:** We thank the reviewer for their thoughtful suggestion. Viral dose information has been included in the respective figure legends.

2. lines 135-140 belong in the intro or discussion.

**Response 2:** For ease of understanding and continuation from the previous result which shows CD40L expression in WT mice, we have included a one-line introduction followed by the result obtained in Figure 1E using CD4-/- mice. This was necessary to introduce that the next set of experiments were conducted in another knockout strain i.e., CD40L-/-.

3. The intro part of the abstract (lines 17-21) and should be rewritten

**Response 3:** Changes have been made in the abstract. (Page 1 Line 17-20)

“Neurotropic mouse hepatitis virus (MHV-A59/RSA59) infection in mice induces acute neuroinflammation due to direct neural cell dystrophy which proceeds with demyelination with or without axonal loss, the pathological hallmarks of human neurological disease Multiple sclerosis (MS).”

4. Mock-infected controls in the flow data would be helpful to show increase/decrease in cell populations. Additionally, was a live/dead stain used to identify cells? What is the large negative cell population? Is that cellular debris?

**Response 4:** The MI control results have been added in the graphs of Figures 4, 5, 6 and 7, as suggested by the reviewers. Due to the area constraint in the figure, we have added the mock data in the graph, but dot plots have been shown only for the infected sets. If reviewers suggest, we can add the mock dot plots in the supplementary figures. (Figures 6,7, 8 and 9 in the revised manuscript)

Live/dead stain was not used, and live cell population was selected based on forward scatter (FSC) and side scatter (SSC) in FlowJo. The large negative population is not cell debris as the cells were selected from singlets followed by live, these represent CD45-negative cell populations in the CNS and CLN. The reference for using FSC/SSC has been added in the revised main manuscripts.

5. The macrophage population was based on CD45 high, CD11b high - this population could also contain neutrophils. This should be mentioned.

**Response 5:** We thank the reviewer for their suggestion. In our original flow data, we already had Ly6G in the myeloid cell panel, so we re-investigated the raw data and considering Ly6G marker and plotted macrophage as CD45hi CD11b+Ly6G-. In our new set of experimental data, we have also curated the CD45hi CD11b+Ly6G- negative population as ‘macrophages’. Both the previous and new data have been compiled together in Figure 6, and 7 in the revised manuscript.

6. Magnification of axon blebbing (Fig. 8A) should be a higher resolution to observe.

**Response 6:** The magnified image (40X) of PLP staining demonstrating axonal blebbing has been added in Figure 11A of the revised manuscript. (Page 9 Line 306)

7. PLP staining was hard to discriminate from background stain in Fig. 8A

**Response 7:** We have added a magnified image (40X) for the PLP staining highlighting the loss of PLP in the lateral white matter tracts in the brain stem regions. (Figure 11A, Page 9 Line 306)

If the images are still not clear, we can remove them as we have LFB stained sections already.

**Reviewer #3:**

1. Line 185: Please correct the figure number to figure 4 (CX3CR1)

**Response 1:** We thank the reviewer for pointing this out. We have made the correction to Figure 6 in the revised manuscript.

Thank you very much again. I look forward to your favorable consideration.

Best regards,

Jayasri Das Sarma.
