# Supplementary material for: CD40L protects against mouse hepatitis virus-induced neuroinflammatory demyelination
Source: PLoS Pathog. 2021 Dec 13;17(12):e1010059. doi: 10.1371/journal.ppat.1010059 (PMC8699621; doi:10.1371/journal.ppat.1010059)
Supplement: Attachment — Submitted filename: Response to Reviewers_PPATHOGENS-D-21-00896R1_11.10.2021.docx [file ppat.1010059.s009.docx]

Dear Editors,

PLOS Pathogens.

We would like to thank you for your kind comments and for further considering our manuscript for publication in Plos Pathogens upon modifying the manuscript according to reviewers' recommendations. We also want to take this opportunity to thank you for sending us the Reviewers comments.

We are grateful to the Reviewers for their appreciation of our revised manuscript. We are also thankful for their further thoughtful comments on our manuscript, which we believe will make it stronger. As per the editorial comments and the reviewers' comments, we have incorporated the changes in the revised manuscript. In the following sections, we have provided point-by-point responses to the issues raised by the reviewers.

**Response to Reviewer's Questions**

**Part I – Summary:**

**Reviewer #1:** I think the experiments provide an excellent characterization of the response of CD40L deficient mice to IC infection with MHV-A59. The authors infer but do not explore the mechanisms involved.

**Reviewer #2:** the authors have addressed all of my concerns. Nice job on the manuscript.

**Reviewer #3:** In this revised manuscript, Saadi et al. have addressed most of the concerns raised by the reviewer. Despite these additions, the revised manuscript is not that well written and has too many main figures.

**Part II – Major Issues: Key Experiments Required for Acceptance:**

**Reviewer #1:** (No Response)

**Reviewer #2:** (No Response)

**Reviewer #3:** The manuscript contains too many main figures. Several figures can be moved to the supplementary section like Figs 2,4, 8,10 and 13.

We thank the reviewer for their thoughtful comments.

As per the reviewer's suggestion, we agree to move Figures 2, 4, 8, and 10 to the supplementary section as Figures S1, S2, S4, and S6, respectively. But given the significant persistence of RSA59 on day 30 p.i. in the brains and spinal cords in the CD40L deficient mice, we decided to keep **Figure 13** (Figure 9 in the newly revised manuscript) in the main manuscript. But still, if the reviewer believes this figure should also go to the supplementary section, we will respect the reviewer's decision and move it to the supplementary section.

**The total figures in the main manuscript now stand at 9 and 7 in the supplementary.**

**Part III – Minor Issues: Editorial and Data Presentation Modifications**
Please use this section for editorial suggestions as well as relatively minor modifications of existing data that would enhance clarity.

**Reviewer #1:** Please check the manuscript for typos and be sure you define all the acronyms the first time.

We thank the reviewer for pointing out our typos, and we apologize for the same. We have carefully checked the manuscript for typological errors and tried to define all acronyms suitably as appropriate.

**Reviewer #2:** (No Response)

**Reviewer #3:**

1. Line 232. On day ?

**Response-1:** In the revised manuscript, suggested changes have been made. The experiment was performed on day 10 p.i. It has been mentioned in the main manuscript. (Line 237, marked-up copy)

2. Line 290: The paragraph should mention that this study assesses day 30 p.i. in the beginning of the paragraph.

**Response-2:** Suggested addition has been made in the revised manuscript. (Line 294, marked-up copy)

3. Line 371 word spacing

**Response-3:** Suggested editing has been made in the revised manuscript. Also, the entire manuscript has been checked for spacing carefully.

4. Line 372: RSA59 induced (n missing)

**Response-4:** Suggested correction has been made in the revised manuscript.

5. Fig 1 C: There is less actin in the Mock infected control suggesting a loading issue. This figure should be repeated or removed.

**Response-5:** We went through all the immunoblots of the biological triplicates and in the revised manuscript, we have replaced with another representative blot. However, if the reviewer still feels the blot should be removed, we will respect the decision and remove the blot from the figure.

6. Fg 12: A-C: At what point was the spinal cord of 5-week old mice assessed? day 30 p.i?

**Response-6:** Yes, the mice were assessed on day 30 p.i. (peak of chronic phase demyelination), it has been mentioned in line 275. In the revised manuscript, we have mentioned the day of the experiment again in Line 294, marked-up copy.

Thank you very much again. I look forward to your favorable consideration.

Best regards,

Jayasri Das Sarma.
